# Death Caused by Water-melon Seed

**Published:** 1855-01

**Authors:** B. Rohrer

**Affiliations:** Germantown, Penna.


DEATH CAUSED BY WATER-MELON SEED. By B. Rohrer, M. D., of Germantown, Penna.—Major W. of Columbia, Penna., a man of strong and robust constitution, blessed with digestive organs of great power, was addicted to drinking, and being a natural gormandizer, he would swallow any thing placed before him, regardless of the consequences. Fish in particular, a favorite dish of his, he would devour bones and all. During the months of May and June, 1852, he frequently hailed me in the street, stating that he had violent cutting pains in his bowels, but as there was no constitutional disturbance, I simply directed him to take a dose of oil. A few weeks after this, he presented me with the cause of all his troubles; he said he felt something working gradually lower and lower down in the rectum, until at last he could reach it with his fingers and remove it. It was a thin hard bone, 1 inch in length, and $\frac{3}{4}$ inch in width; its edge slightly serrated, and very sharp; whilst it was passing along the rectum, he had frequent discharges of blood; it was supposed to be the side bone of the head of a catfish.

On the 12th of September, upon my return to the office, I found a message from the major. I immediately called, but was informed that he had been complaining all morning of colic, and had just left in the train of cars, to visit his niece some distance in the country. I heard nothing more of him until the 22d, when I was requested to visit him with Dr. Jones, of Bainbridge. When we arrived, he was delirious, muttering to himself—pulse frequent, irregular, small and wiry; abdomen much distended, and tense as a drum; vomiting a vitiated mucus, commixed with bile; extremities cold—death soon followed. Had no evacuations from the 12th up to his death, although all the usual means had been employed.

*Post-mortem 36 hours after death.*—Abdominal cavity containing a quantity of offensive gas and fecal matter. Gangrene of the ileum near the ileo-cæcal valve, containing 20 water-melon seeds, with the slippery surface destroyed, and adhering firmly to each other, making a strong ball. Also, gangrene of the sigmoid flexure containing over 100 seeds, forming an exceeding hard ball.—*Am. Jour., Med. Sciences.*

